# Potentiometric Water Sensor For Eutectic Solvents Containing Choline Chloride and Ethylene Glycol

**DOI:** 10.1021/acsomega.5c03135

**Published:** 2025-07-07

**Authors:** Hayder A. S. Al-Jaafari, Jennifer M. Hartley, Molly E. Keal, Andrew P. Abbott, Jake M. Yang

**Affiliations:** † School of Chemistry, 4488 University of Leicester, University Road, Leicester LE1 7RH, U.K.; ‡ Department of Al-Najaf Education, General Directorate of Education in Al-Najaf, Ministry of Education, Al-Najaf 54001, Iraq

## Abstract

Electrochemical reprocessing of metals using eutectic solvents (ESs) is a promising approach due to its availability, cost-effectiveness, ease of preparation, and environmental benefits. However, a significant challenge associated with ESs is their tendency to absorb moisture from the atmosphere, which can impede and alter electrochemical recovery processes. Here, we examine a novel electrochemical method for quantifying the amount of water content absorbed by a commonly used ES formed from choline chloride and ethylene glycol (ChCl:2EG). ChCl:2EG is exposed to the open atmosphere for over 8 days with Cu^2+^ ions initially present at 10 mM. By simply analyzing the relative reduction potentials of the Cu^2+/+^ redox couple against the Cu^+/0^ redox couple, formed in situ in the voltammetry sweep, the water content of the ES adsorption from the atmosphere was determined via a calibration plot obtained using external reference samples. An upper maximum of ca. 35% water was observed over the 8 days of atmospheric exposure. Instead of depending on the absolute potential measured against a reference electrode, the analysis has measured the potential difference between the two redox couples in situ in solution. As a result, it is more user-friendly, as the measurement is independent of the choice of the reference electrode. We foresee that this rapid in situ potentiometric approach in determining water content in ChCl:2EG could be generalized similarly for other ESs.

## Graphical abstract

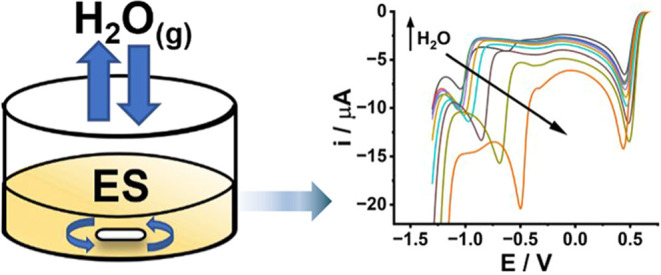

## 1 Introduction

Eutectic solvent (ES) is a developing class of environmentally friendly solvents that share similar properties with ionic liquids (ILs), such as high thermal stability, low volatility, low vapor pressure, and adjustable polarity.^1^ However, unlike ILs, which can be costly, toxic, and lacking biodegradability, ESs are often affordable, biodegradable, nontoxic, and easier to synthesize and have attracted significant interest for their potential as economical and sustainable solvents.^2,3^

ChCl:2EG, a well-studied ES composed of choline chloride (ChCl, a hydrogen bond acceptor) and ethylene glycol (EG, a hydrogen bond donor) in a 1:2 molar ratio, is widely used in research due to its ease of preparation, nontoxicity, and representative ES properties.^4–6^ We note that, although commonly referred to as a “ES” in literature, ChCl:2EG is not truly “deep” when compared to predictions from ideal solution theory.^7^ A major challenge associated with ChCl:2EG is its strong hygroscopic nature, causing it to absorb moisture from the atmosphere.^8^ Water adsorption significantly impacts the physicochemical properties of ES as a solvent (viscosity, conductivity, density) and also the performance of electrochemical reprocessing of metals.^9^ Critically, the presence of water promotes the formation of oxides or hydroxides on the metal surface, creating a passivation layer that inhibits further electrochemical reactions, reduces recovery efficiency, and increases energy consumption.^10^ These issues are especially problematic in industrial applications, where high process efficiency and product purity are critical. With that said, the presence of small amounts of water in ESs can be beneficial in some cases, such as the electroplating of copper or the dissolution of gold, as the overall rate at the electrode–solution interface is typically limited by the rate of mass-transport.^11^ In large-scale industrial applications involving ESs, where moisture-free atmospheres are neither practical nor economically viable, it is crucial to have techniques that can routinely monitor and therefore control the water content at the desired level.^12^

One of the most commonly employed techniques for water quantification is the Karl Fischer (KF) coulometric titration.^13^ While KF is widely used due to its high sensitivity and selectivity, this technique presents certain limitations, particularly in the context of ES. This method relies upon the redox reaction between I_2_, sulfur dioxide, and water. However, in certain ES systems, such as ChCl:2EG, iodine can exist as I_2_Cl^–^/ I_3_^–^,^14^ which can interfere with the redox process and affect the accuracy of the measurement. Furthermore, the KF titration can lead to the formation of precipitates in some Type IV ES,^15^ further limiting its applicability as a general water quantification method in ESs.

In contrast, our method has been developed to be specifically used in ES systems and uses copper chloride, which is low-cost and readily available. The key advantages of the potentiometric water sensor include its simplicity, minimal sample preparation, and the measurement being able to be conducted directly in the electrochemical cell. However, in this method, there is still some exposure to ambient conditions, which may introduce a risk of moisture gain or loss during the measurement.

This study focuses on the development of a potentiometric electrochemical probe that allows the water content in a commonly used ES, ChCl:2EG, to be determined. Here, copper ion redox couples (Cu^2+/+^ and Cu^+/0^) are employed as an electrochemical probe to determine the water content of ChCl:2EG. In the absence of water, Cu^2+^ ions in ChCl:2EG are solvated by chloride ions and exist predominantly in the forms of [CuCl_4_]^2–^ and [CuCl_2_].^16^ However, the introduction of water leads to the coordination of water ligands to copper ions to form mixed chloride-aquo species such as copper­(II) chloride tetrahydrate [CuCl_2_(H_2_O)_4_] and copper­(II) hexahydrate [Cu­(H_2_O)_6_]^2+^ in high water levels.^10,17^ In this work, we reveal that the reduction potential between Cu^2+/+^ and Cu^+/0^ is linearly proportional to the level of water content present in ChCl:2EG. By recording cyclic voltammetry of millimolar concentrations of Cu^2+^ present in ChCl:2EG, we determined the amount of water in ChCl:2EG adsorbed from the atmosphere over 8 days of atmospheric exposure. As far as the authors are aware, this is the first potentiometric approach to measure water content in ES.

## 2 Material and Methods

### 2.1 Chemicals

The deep eutectic solvent made from choline chloride and ethylene glycol, referred to hereafter as ChCl:2EG, was synthesized by mixing choline chloride (ChCl, 99% pure, Sigma-Aldrich) with ethylene glycol (EG, 98% pure, Sigma-Aldrich) in a 1:2 molar ratio at 50 °C until a homogeneous clear liquid was obtained. The ChCl:2EG was then heated at 60 °C with continuous stirring for 24 h to minimize moisture content. Afterward, the solvent was cooled to room temperature in a sealed glass bottle.

Solutions of 10 mM anhydrous copper chloride (CuCl_2_, 98% pure, Fluorochem, U.K.) were prepared separately in both ChCl:2EG and deionized water. Various weight percentages (0–50 wt %) of the aqueous solution containing 10 mM Cu^2+^ were added to the ChCl:2EG solution to investigate the effect of water content on the voltammetric behavior of Cu^2+^. It should be noted that the final concentration of Cu^2+^ in the resultant ES–water mixture remained unchanged at 10 mM.

### 2.2 Measurement of Initial Water Content Using Karl Fischer Titration

The initial water content of the ChCl:2EG solution after synthesis following the procedure described above was 2.1 wt % as determined using Karl Fischer coulometric titration (Eco Coulometer with generator electrode without diaphragm, Metrohm, Switzerland). 1 mL of the sample was injected into the titration cell containing a premixed Karl Fischer reagent until measurement was established.

### 2.3 Cyclic Voltammetry

Electrochemical measurements were conducted using a potentiostat (Metrohm Autolab PGSTAT204, Netherlands, controlled using Nova 2.1 software) equipped with a three-electrode system. A glassy carbon disk-electrode (MF-2012 BASi) with a diameter of 3 mm was employed as the working electrode, while a platinum flag electrode (in-house made) with an average surface area of 64 mm^2^ served as the counter electrode. The reference electrode was composed of a silver wire immersed in a 90 mM solution of AgCl in ChCl:2EG, housed in a fritted electrode body. Cyclic voltammetry (CV) was performed on each of the ES:water solutions with a known water content. The solutions were thermostated to 298 K prior to CV measurements. The potential window for the cyclic voltammetry measurements was set from +0.88 to −1.32 V (which includes the redox process of Cu^2+^/ Cu^0^) vs the 90 mM Ag/AgCl in ChCl:2EG reference electrode, and the voltage scan rate was maintained at 10 mV s^–1^. The working electrode was polished before each measurement with alumina (BUEHLER, Germany) of decreasing particle sizes (1.0, 0.3, and 0.05 μm) and rinsed with deionized water. The electrode was dried with warm air using a hairdryer.

To investigate the effect of exposure to atmospheric moisture on the copper chloride, a solution of 10 mM CuCl_2_ in dry ChCl:2EG was exposed to the ambient laboratory atmosphere for 8 days using a wide crystallizing dish (11 cm diameter). During this period, the solution was constantly stirred and thermostated to 298 K to minimize the effect of laboratory temperature variation overnight on the absorption of moisture. At a series of time intervals (0, 0.25, 0.5, 1, 2, 4, 8, 24, 48, 72, 96, 120, 144, 168, and 192 h), a cyclic voltammogram of this solution was obtained. The potential difference (Δ*E*) and peak current (*I*_p_) values of the copper redox couples as the ChCl:2EG solution was exposed to the atmosphere were analyzed and reported herein. It was observed that the volume of the ChCl:2EG solution was significantly increased after prolonged exposure to air.

### 2.4 UV–vis Spectroscopy

UV–vis spectroscopy was conducted using a Mettler Toledo UV5 Bio spectrometer using the same 10 mM CuCl_2_ in ChCl:2EG solutions with varying amounts of water as used for the voltammetry experiments. The cuvettes used were 0.1 mm quartz glass slides due to the intense coloration of the copper complexes in solution.

## 3 Results and Discussion

This work focuses on estimating the water content in ChCl:2EG using the electrochemical behavior of the Cu^2+^ ions in solution. First, the work explores the reduction of Cu^2+^ and Cu^+^ in ChCl:2EG systems with different amounts of added water. Having understood how the presence (or absence) of water affects the reduction potential of Cu^2+^ and Cu^+^, the focus then turns to producing a calibration curve relating the reduction potential between the two copper redox couples (Δ*E*) to the water content of ChCl:2EG. Using the calibration curve, the amount of water adsorbed by the ChCl:2EG solvent during 8 days of air exposure was determined.

### 3.1 Voltammetry of 10 mM Cu^2+^ in ChCl:2EG and Water (Constant Copper Concentration)

Figure 1 shows CV measurements for solutions containing 10 mM CuCl_2_ in dry ChCl:2EG and in a 0.1 M aqueous solution of choline chloride (ChCl). Note that the term ‘dry’ herein refers to the *lack of added water* to the synthesized ChCl:2EG solution. The ChCl:2EG solution as synthesized, however, contains 2.1 wt % of water, which was determined independently using a Karl Fischer coulometric titrator at the start of the experiment. In both cyclic voltammetric studies, a glassy carbon (GC) macro-disk working electrode (3 mm diameter) was used. To minimize liquid junction potential, the same solvent was used in the reference electrodes. This also prevents the crystallization of ChCl:2EG in high water media. So, in the case of ChCl:2EG, the applied potential was referenced against 90 mM Ag/AgCl in ChCl:2EG, whereas an Ag/AgCl electrode in saturated KCl was used as the reference for CV measurements in aqueous media. The voltammograms are recorded at a voltage scan rate of 10 mV s^–1^.

**Figure 1:**
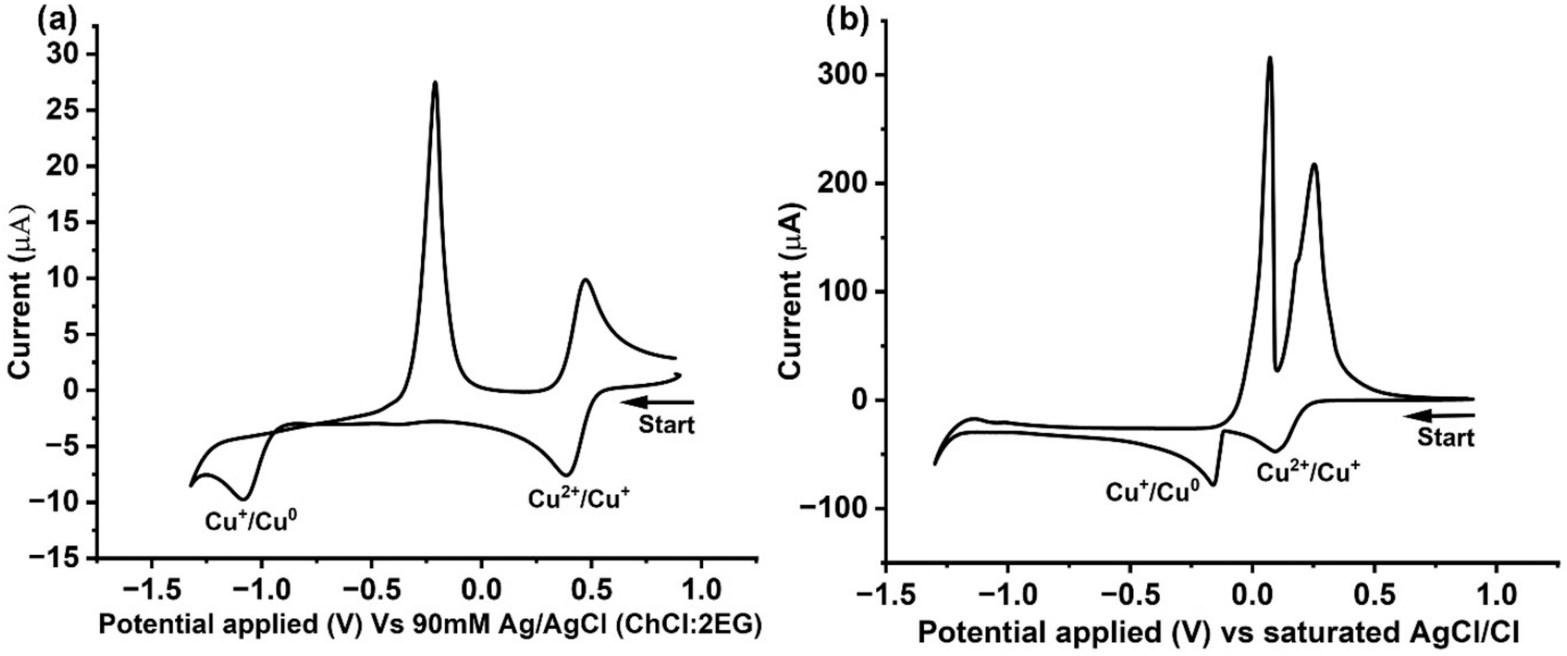
Cyclic voltammograms of 10 mM CuCl_2_ in (a) dry ChCl:2EG and (b) aqueous solution containing 0.1 M ChCl. Note that the ‘dry’ ChCl:2EG solution contains 2.1 wt % water after synthesis. Measurements were made with a voltage scan rate of 10 mV s^–1^ and at 298 K. The working electrode was a GC disk (diameter 3 mm) with a Pt flag counter electrode. The reference electrode was 90 mM Ag/AgCl in ChCl:2EG for the ES system and Ag/AgCl electrode in saturated KCl­(aq) for the aqueous system.

In ChCl:2EG, Figure 1a, the reversible Cu^2+/+^ redox couple was observed at +0.5 V vs the 90 mM Ag/AgCl in ChCl:2EG reference electrode, which was followed by the electrodeposition of Cu^0^ from Cu^+^ below −1.0 V. On the reverse scan, electrochemical oxidation of the deposited Cu_(s)_ during the forward sweep is seen at −0.32 V. The voltammetry profile of CuCl_2_ in an aqueous solution with 0.1 M of ChCl as the supporting electrolyte is, however, very different in comparison, Figure 1b. While two independent redox couples are present in aqueous media, a significant reduction in the redox potential between the two copper redox couples is clearly seen. The first cathodic peak at ca. + 0.12 V vs the Ag/AgCl aqueous reference electrode is due to the reduction of Cu^2+^ to Cu^+^, whereas the second cathodic peak at ca. −0.12 V is the reduction of the formed Cu^+^ to Cu^0^. On the reverse scan, a characteristic stripping peak of Cu^0^ is seen at −0.06 V vs the Ag/AgCl electrode, and the second peak seen at +0.13 V is the one-electron oxidation of Cu^+^ to Cu^2+^. Despite the same concentration of Cu^2+^ initially present in both solutions, the peak reduction and oxidation currents are much higher in aqueous solution than in ChCl:2EG due to the much higher viscosity of ES solvents, which in turn affects the diffusion coefficients of ions in solution.^18^

Clearly, here, the redox potential values of the two copper redox couples relative to each other are different due to the different ligand coordinations and, importantly, different chemical environments. In ChCl:2EG, copper ions are present as a mixture of [CuCl_4_]^2–^ and [CuCl_2_]^−^/[CuCl_3_]^2–^ species, depending on the oxidation state of the copper ions,^16^ while in the aqueous solution containing 0.1 M ChCl, Cu^2+^ is fully hydrated in the form of [Cu­(H_2_O)_6_]^2+^ and Cu^+^ precipitates to form CuCl(s).^10,17^

The difference in the redox potentials relative to each other within a particular solvent can be linked to the relative stabilities of the copper species in solution. In ChCl:2EG, the Cu^2+/+^ and Cu^+/0^ redox couples are separated by ca. 1.0 V, whereas in the aqueous ChCl system, the redox couples are separated by only ca. 0.2 V. This reflects a smaller Gibbs energy difference between Cu^2+^ and Cu^+^ in aqueous solution than in ChCl:2EG solution, likely due to Cu^+^ being less stable in aqueous solution.^10^

Next, cyclic voltammetry experiments were carried out in solutions of ChCl:2EG with different percentages of water, and a fixed concentration of dissolved CuCl_2_ (10 mM), Figure 2b, shows voltammograms of solutions with an overall water content ranging from 2 to 52 wt %, accounting for the initial 2% water present in the ‘dry’ ChCl:2EG solution after synthesis. Note that, for clarity of display, the forward (reductive) sweep of CVs of 25 and 50 wt % water ChCl:2EG solutions are shown separately in Figure 2c. The solutions were allowed to equilibrate prior to the voltammetry experiment by vigorously stirring in a sealed glass container for 60 min, and the solution was thermostated at 298 K prior to the cyclic voltammetry measurement. Identical to voltammograms shown in Figure 1, the voltammograms herein were also recorded at a glassy carbon electrode (3 mm diameter), with a voltage scan rate of 10 mV s^–1^. Within the range of the ES-water mixtures studied, the electrochemical behavior of the copper species, unsurprisingly, more closely resembles that seen in dry ChCl:2EG, as both Cu^2+/+^ and Cu^+/0^ redox couples are present with peak-to-peak separation of ≥ 1.0 V. Interestingly, the electrode potential for the Cu^2+/+^ pair remains unchanged (±20 mV), but the onset potential for the reduction of Cu^+^ to Cu^0^ becomes more anodic with more water added to the ES. Between 0 and 3.2 wt % water, the shift in onset potential is only 30 mV more anodic. Above this value of water content, the onset potential shift is much more pronounced, up to 620 mV more anodic in the 50 wt % water solution. The peak current values can be seen to increase with increasing water content, as expected due to lower solution viscosity, which results in larger diffusion coefficients of the ions involved in the electrochemical reactions.^10,18^

**Figure 2:**
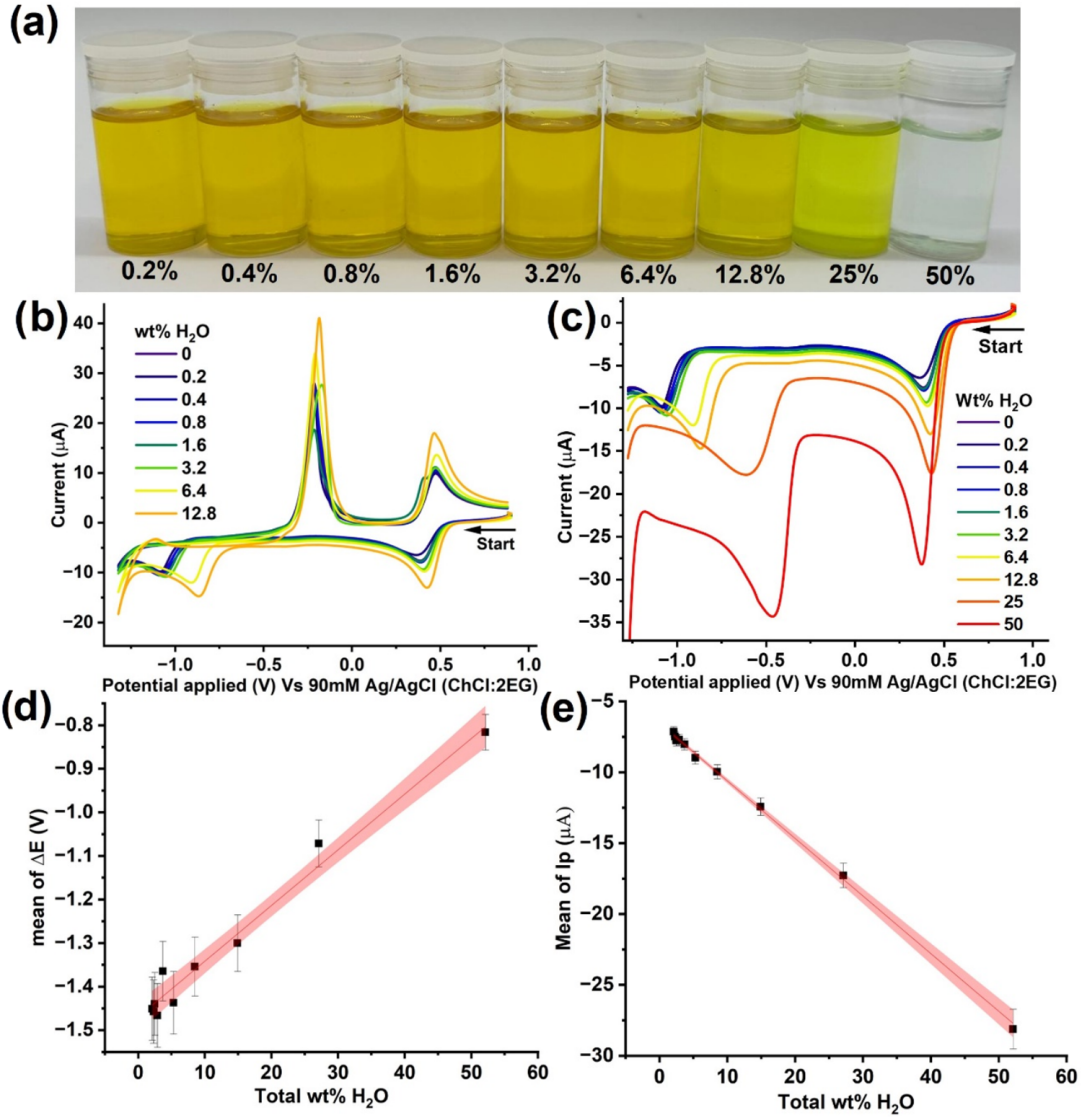
(a) Solutions of 10 mM CuCl_2_ in ChCl:2EG with 10 mM CuCl_2_ in different added water percentages (0.2–50 wt %) and CVs of 10 mM CuCl_2_ in ChCl:2EG with different water percentages: (b) 0.2–12.8 wt %, and (c) 0.2–50 wt %. The reference electrode was 90 mM Ag/AgCl (ChCl:2EG). Both CVs were recorded at a voltage scan rate of 10 mV s^–1^ at 298 K using a GC working electrode (diameter = 3 mm) and a Pt flag as a counter electrode. (d) Mean reductive peak-to-peak separations, Δ*E*, with the total wt percentage of water. (e) Mean peak reductive current, *I*_p_, of the Cu^2+/+^ redox couple with the total wt percentage of water. The sample size for (d) and (e) is three.

This change in onset potential with higher water content can be linked to the change in relative concentrations of water and chloride in solution, resulting in the formation of different copper chloride-aquo complexes depending on the water content within the ES.^10,16^ To investigate this, we conducted UV–vis spectroscopy to examine the effect of water addition on copper­(II) chloride speciation in ChCl:2EG. The resulting spectra are provided in the Supporting Information (Figure S2). For solutions containing 0.2–12.8 wt % added water, three distinct charge-transfer bands are present at 239, 290, and 406 nm, characteristic of the tetrachlorocuprate­(II) complex [CuCl_4_]^2–^. At 25 wt % water, the peaks at 239 and 290 nm broaden, and at 50 wt % water, the two peaks merge, although this does not indicate a significant change in Cu­(II) speciation. This is consistent with those reported in the literature; for example, in a 50 mM CuCl_2_·2H_2_O in ChCl:2EG with added water, the UV/vis spectra indicated that up to 40 wt % the dominant species remained [CuCl_4_]^2–^ with no significant changes to speciation to that of pure ChCl:2EG.^11^ A further EXAFS and UV–vis study by De Vresse et al. on an analogous choline chloride/CuCl_2._2H_2_O ES system confirmed the dominant species is [CuCl_4_]^2–^ up to 39 wt % water.^17^

From a combination of these potential differences and the current magnitude of the reduction signals of Cu^2+^, one might be able to determine the amount of water present in ChCl:2EG.

Figure 2d plots the measured peak-to-peak separation (Δ*E*) during the forward scan as a function of the total water wt % percentage (accounting for the initial 2%). As can be seen from Figure 2c, the linear relationship between the reductive potential difference of copper, Δ*E*, against the total weight percentage of water (Total wt % H_2_O) is ideal as a potentiometric sensor for water content in ES. The red-shaded region shows the 95% confidence band.

Figure 2e plots the reduction peak current of the Cu^2+/+^ redox couple, *I*_p_ (A), against the total wt % of H_2_O. A linear trend was also seen. This suggests that as the water content rises from near zero to approximately 50 wt %, the system experiences a pronounced increase in the magnitude of the reduction current arising from Cu^2+^ + e^–^ → Cu^+^. This is in full agreement with the decrease in viscosity of the ES solution with increasing water content, which inevitably increases the diffusion coefficient of Cu^2+^.^10^

We note, however, in reality, a ChCl:2EG solution containing an initial fixed concentration of cupric ions exposed to a “wet” atmosphere will expand in volume as it absorbs water from air. Therefore, both the electrolyte ChCl and cupric ions will be diluted. The effect of decreasing concentration of cupric ions with added water into a ChCl:2EG solution initially containing 10 mM Cu^2+^ is investigated in the next section.

### 3.2 Varying the Concentration of Cu^2+^ with Water Dilution

Similar to that above, CV experiments were performed in ChCl:2EG solutions containing an initial concentration of 10 mM CuCl_2_ in varying water percentages. Different from the above, however, the addition of water, in this case, into the ChCl:2EG solution results in the dilution of both the electrolyte and cupric ions from the initial 10 mM concentration. Voltammograms shown in Figure 3a depict the electrochemical response of Cu^2+^ containing ChCl:2EG solutions with total water content ranging from 2 to 52 wt %. To enhance the clarity of display, the reductive (forward) sweeps of the CVs for 0 to 50 wt % of added water in ChCl:2EG are displayed separately in Figure 3b. Before recording the voltammograms, the solutions were equilibrated by stirring in a sealed container for 60 min and maintained at 298 K. Aside from the diluting cupric ion concentration with water, the experimental conditions were the same as above. The total water content in the system varied from 2 to 52 wt %, considering that the synthesized ‘dry’ ChCl:2EG solution initially contained 2.1 wt % water. The voltammograms of varying Cu^2+^ concentrations with water dilution, shown in Figure 3a, show trends similar to those seen where [Cu^2+^]_bulk_ was fixed at 10 mM, as shown in Figure 2a. The electrode potential of the Cu^2+/+^ redox pair remains largely unchanged (within ± 20 mV), but the Cu^+^ to Cu^0^ reduction peak potential shifts anodically as the water content increases. The only notable difference is that the observed reductive peak currents are smaller, which is reasonable because of the interplay between a reduction of [Cu^2+^]_bulk_ with dilution and the increase in the diffusion coefficient of Cu^2+^ in a less viscous ‘wet’ ionic medium.^10^

**Figure 3:**
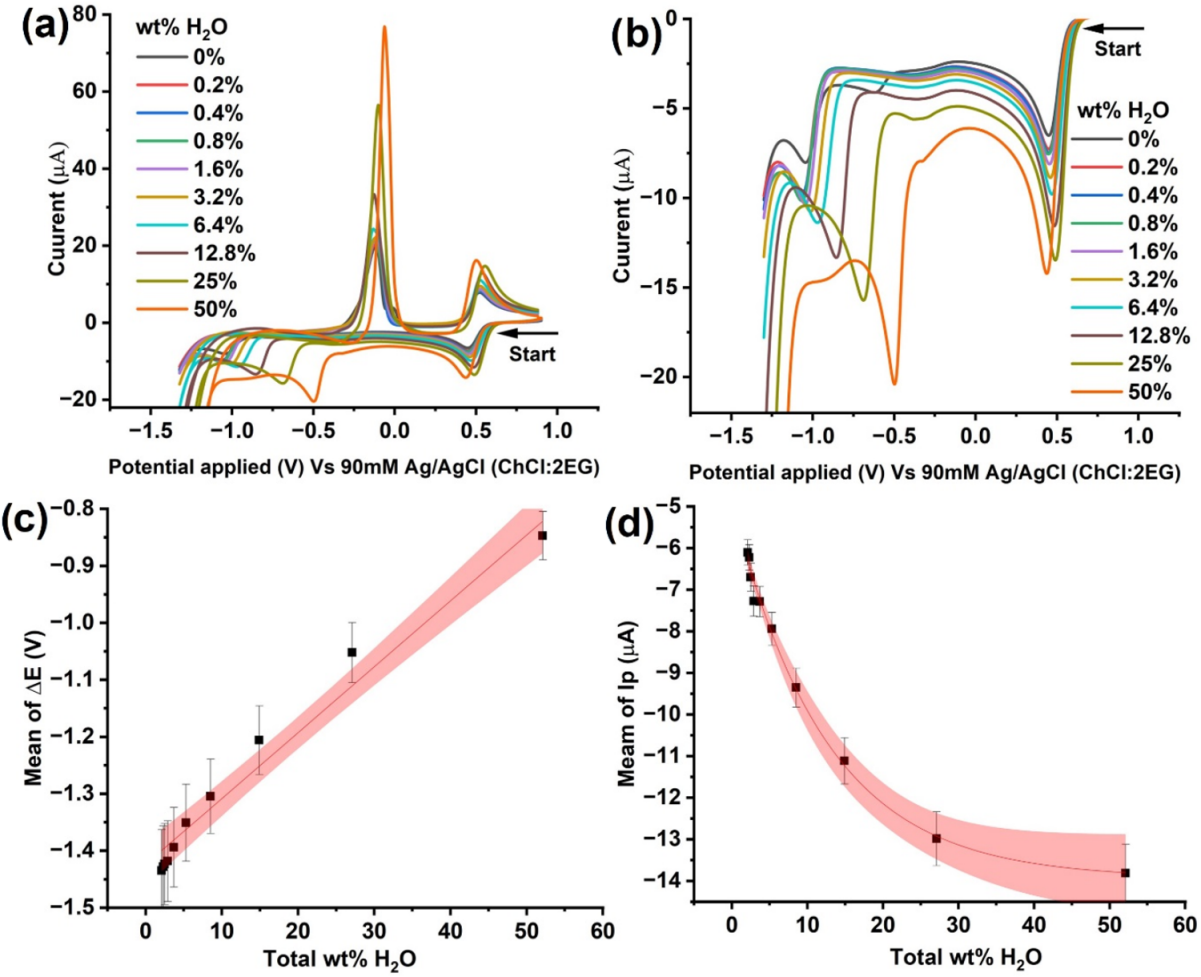
CVs of (a) 10 mM CuCl_2_ in ChCl:2EG with different percentages of water (without CuCl_2_) vs 90 mM Ag/AgCl (ChCl:2EG) and (b) zoomed-in CVs from Figure 3a highlighting the cathodic peaks for clarity. All CVs were recorded at a voltage scan rate of 10 mV s^–1^ at 298 K using a GC working electrode (diameter = 3 mm) and a Pt flag as a counter electrode. (c) Mean reductive peak-to-peak separations, Δ*E*, with total wt percentage of water. (d) Mean peak reductive current, *I*_p_, of the Cu^2+/+^ redox couple with the total wt percentage of water. The sample size for (c) and (d) is three.

Figure 3c plots the peak-to-peak separation (Δ*E*) measured during the forward scan with respect to the total weight percentage of water, accounting for the initial 2 wt % water. The observed linear trend between Δ*E* and wt % H_2_O, similar to the case for constant 10 mM of Cu^2+^ shown in Figure 2c, suggests an application for a potentiometric sensor for water content in ES. The red-shaded area represents the 95% confidence band. On the other hand, the variation of the reduction peak current for the redox couple, Cu^2+/+^, shown in Figure 3d, presents a nonlinear trend. This is due to the interplay between a reduced ES viscosity upon water addition,^18,19^ which enhances the diffusion coefficient of Cu^2+^ but, at the same time, decreases [Cu^2+^] in the bulk. Note that Figure S1 shows similar results if one corrects the peak current measured under a constant 10 mM of [Cu^2+^]_bulk_, shown in Figure 2d, which shows a hypothetical dilution of copper concentration with added water. See SI Section S1 for full discussions. Based on these calibration plots developed using Δ*E* and *I*_p_, the work next turns to recording cyclic voltammograms of a ChCl:2EG solution exposed to the open atmosphere over a period of 8 days.

### 3.3 Estimating the Water Content of ChCl:2EG Exposed to the Atmosphere

A solution of 10 mM CuCl_2_ in dry ChCl:2EG was prepared and exposed to an open atmosphere over 8 days. The solution is constantly stirred to ensure that any moisture absorbed is homogeneously distributed within the viscous ES. A schematic of the setup is shown in Figure 4a. The cyclic voltammetry measurements were conducted at different time intervals since the exposure using the same electrodes and conditions as described above.

**Figure 4:**
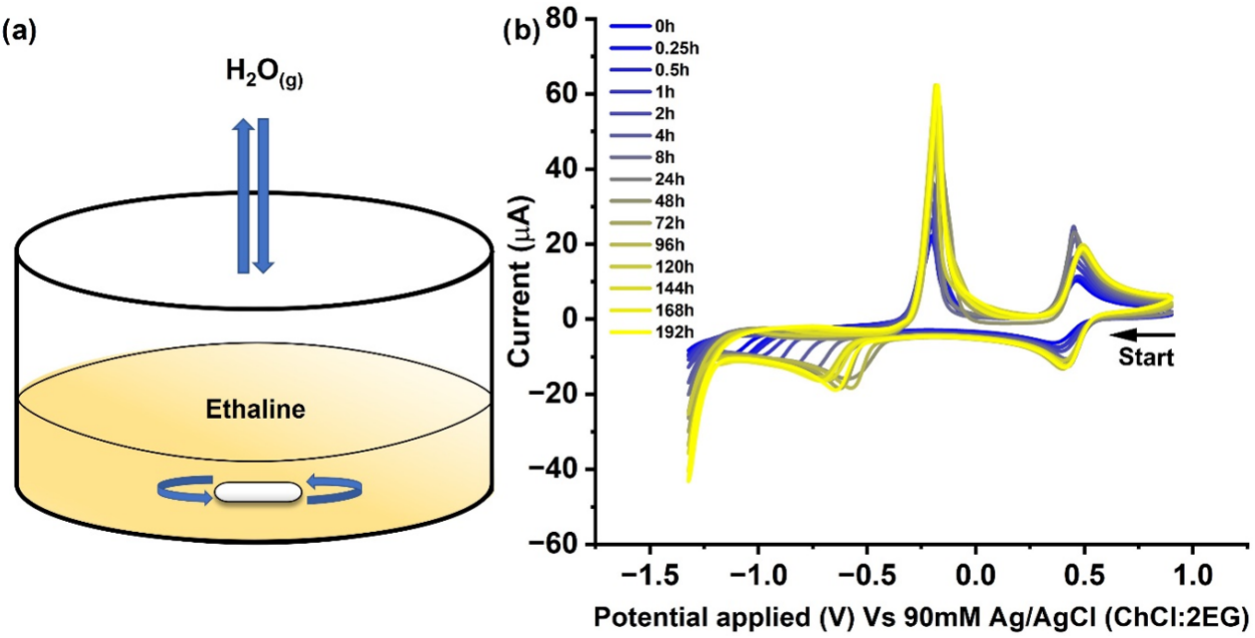
(a) Schematic of the experimental setup for atmospheric moisture absorption and (b) voltammograms of 10 mM CuCl_2_ in ChCl:2EG after exposure to the atmosphere over 192 h. The voltammograms were recorded at a GC electrode (diameter 3 mm), with a voltage scan rate of 10 mV s^–1^ and a temperature of 298 K. The counter electrode was the Pt flag, and the reference electrode was 90 mM Ag/AgCl (ChCl:2EG).

Figure 4b shows a series of CVs recorded at different air exposure times, ranging from 0 h (blue curve) to 192 h (yellow curve) of exposure. Over time, water vapor from the atmosphere is absorbed into the ChCl:2EG, resulting in a significant increase in volume notable by the eye. As the exposure time increases, the peak current values for both redox couples increase only slightly, as can be seen in Figure 4b, and the peak-to-peak potential difference between the redox couples during the forward sweep was seen to decrease. The current behavior, as expected and discussed in the above section, is a direct result of increasing water content within the ES, which, in turn, decreases the solvent viscosity and dilutes the concentration of analyte, Cu^2+^.^10^

Figure 5a plots the reductive peak-to-peak potential as a function of the exposure time measured from CVs shown in Figure 4b. This inference of water wt % from the Δ*E* calibration plots is plotted in Figure 5b. As can be clearly seen, increased exposure time of ChCl:2EG as made resulted in increasing water absorption up to a maximum of 35 wt %. Excellent agreement was seen between the two Δ*E* calibration plots: fixed 10 mM [Cu^2+^]_bulk_ (shown in Figure 2d) and diluting [Cu^2+^]_bulk_ with added water (Figure 3c). The authors hypothesize that the fluctuations in the water wt % in ES after 50 h of air exposure are likely due to a fluctuation of atmospheric moisture, which varies from day to day.

**Figure 5:**
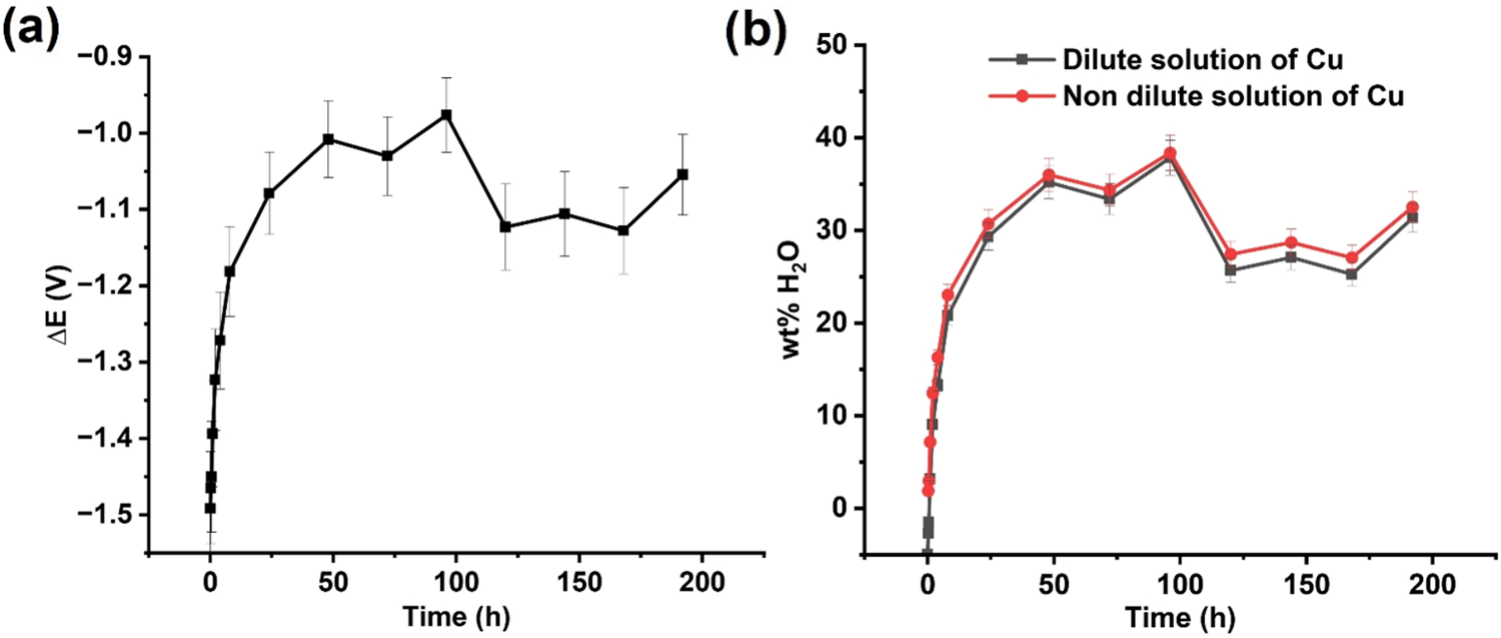
(a) Variation of Δ*E* over time and (b) calculated water weight percentage in ChCl:2EG after air exposure at different times, determined using Δ*E* calibration curves from Figures 2c and 3d.

Figure 5b shows excellent agreement between results inferred from constant 10 mM [Cu^2+^]_bulk_ and varying [Cu^2+^]_bulk_ calibration plots. Counterintuitively, one might think there should be a difference between the black and the red curves since the standard formal potential for the Cu^+/0^ redox couple is pinned by the concentration of Cu^+^ at equilibrium since the activity of Cu^0^ is unity. However, the dilution factor when 50 wt % of water is added to the ChCl:2EG solution (density = 1.07 g cm^–3^)^20^ is only approximately 2. Let us assume, for simplicity, that the potential-determining concentration of Cu^+^ formed near the electrode is decreased similarly by a factor of 2; then, the reduction peak potential of Cu^+/0^ is expected to shift cathodically by ca. 18 mV (= 59 mV × log(0.5)). Notice that 18 mV is negligible compared to the 500 mV in Δ*E* at 50 wt % water.

Similarly, one could infer water content using the Cu^2+^ reduction peak current calibration curve. The results are shown in Figure S2. The inference of H_2_O wt % from current calibration plots (amperometric) averages around 30 wt % of H_2_O at prolonged air exposure, which agrees with the potentiometric approach as seen above. But we note that the wt % water inferred using the amperometric approach is highly varied as compared to the potentiometric approach. This is likely due to the fact that the absolute reduction peak current of Cu^2+^ is highly sensitive to the exact distribution of cupric ions ligand coordination at equilibrium; this is commonly seen for redox couples with overlapping redox potentials where a small perturbation of, for example, electron transfer rate constants and concentrations could lead to a significant change in the measured apparent peak current.^21^

## 4 Conclusions

This study investigates the influence of atmospheric water absorption by ChCl:2EG using readily available Cu^2+^ salts. The highly hygroscopic nature of ChCl:2EG poses a significant obstacle for wide industry applications due to how the water adsorption from the atmosphere could impact the electrochemical property of the solvent. In this work, we have developed a potentiometric sensor for the water content of ES by measuring the peak-to-peak separation of the Cu^2+/+^ and Cu^+/0^ redox couples in the range of 0–50 wt % added water content. Note that 2.1% of water was present in the just synthesized ES as inferred from Karl Fischer titration, so the detection limit of this technique is a minimum of 2.1%.

Increased water content in ChCl:2EG was found to induce a significant anodic shift in the Cu^+/0^ reduction peak by ca. + 620 mV with 50 wt % of added water compared to ‘dry’ ES. This shift was attributed to the change in ligand solvation around cuprous ions from chloride-rich coordination to water-rich. The change in the solvating ligands with increasing water content did not seem to affect the reduction potential of the Cu^2+/+^ redox couple. This is not unexpected since the electron transfer mechanism for Cu^+/0^ is likely the inner sphere involving the removal of ligands prior to the formation of Cu(s) on the surface of the electrode, whereas the electron transfer mechanism for Cu^2+/+^ is the outer sphere.

This research proposes a new potentiometric sensing method to estimate water contents in ES. We have shown that the results obtained are unaffected by the dilution of the analyte, Cu^2+^, initially present in the ES medium over the concentrations studied herein. These findings provide a new analytical tool for monitoring the ‘wetness’ of ES for industrial applications. The validity of this method could potentially be further deployed in a range of different ES compositions, and this may be of value for monitoring carboxylic acid-based ESs that slowly esterify over time.

## Supplementary Material



## Notes

Calibration plot of peak current vs total wt % water over 192 h; UV–vis spectra of copper­(II) chloride dissolved in ChCl:2EG with varying water contents; and water content based on peak current calibration plot (PDF)
