# Supplementary material for: Potentiometric Water Sensor For Eutectic Solvents Containing Choline Chloride and Ethylene Glycol
Source: ACS Omega. 2025 Jul 7;10(28):30843–50. doi: 10.1021/acsomega.5c03135 (PMC12290665; doi:10.1021/acsomega.5c03135)
Supplement: Supplementary file 1 [file ao5c03135_si_001.pdf]

Supplementary Information:
Potentiometric water sensor for eutectic solvents containing choline chloride and ethylene glycol

Hayder A. S. Al-Jaafari,[a, b] Jennifer M. Hartley,[a] Molly E. Keal,[a] Andrew P. Abbott,[a] Jake M. Yang[a*]

[a] *School of Chemistry, University of Leicester, Leicester, LE1 7RH*

[b] *Department of Al-Najaf Education, General Directorate of Education in Al-Najaf, Ministry of Education, 54001, Al-Najaf, Iraq*

[*] *Corresponding author. Email address: jake.yang@leicester.ac.uk*

SI Section 1

The peak reduction current of Cu^{2+} in ChCl:2EG solutions containing different wt% of water and a fixed $[Cu^{2+}]_{bulk}$ of 10 mM is shown in **Figure 2 e)**. The Randles–Ševčík equation[1] shows a linear relationship between the peak current, I_p, and bulk concentration of Cu^{2+}.

$$I_p = (2.69 \times 10^5)\, n^{3/2}\, A\, [Cu^{2+}]_{bulk}\, D^{1/2}\, V^{1/2}$$

where n is the number of electrons involved in the redox reaction, A is the area of the electrode (cm^2), D is the diffusion coefficient ($cm^2\ s^{-1}$) of Cu^{2+}, and V is the voltage scan rate ($V\ s^{-1}$). Therefore, the peak current of Cu^{2+} in the hypothetical case where the addition of water into the ES, originally containing 10 mM of Cu^{2+}, acts to dilute the $[Cu^{2+}]_{bulk,}$ then the hypothetical concentration-corrected peak current, as shown in **Figure S1**, is expected to decrease with added wt% of water. Note the similarity between the concentration-corrected I_p shown in Figure S1 to that measured experimentally, shown in Figure 3d).

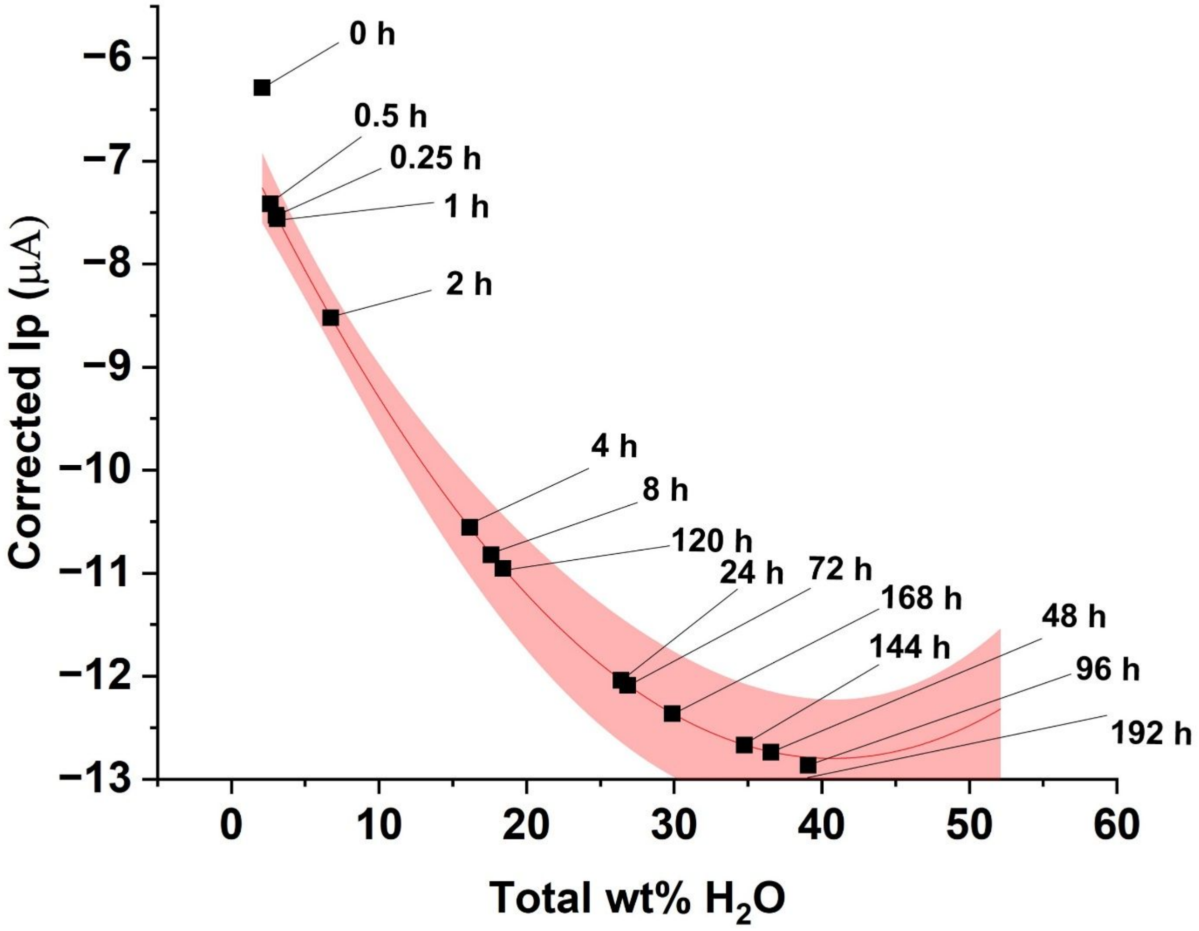

Figure S1: A plot showing the hypothetical peak current of Cu^{2+} if the cupric ions, fixed at 10 mM, were to be diluted with added water content. The original data, shown in **Figure 2e)**, were corrected for a hypothetical concentration dilution as described by the Randles–Ševčík equation. The green dots represent experimental I_p values obtained after different exposure times to air.

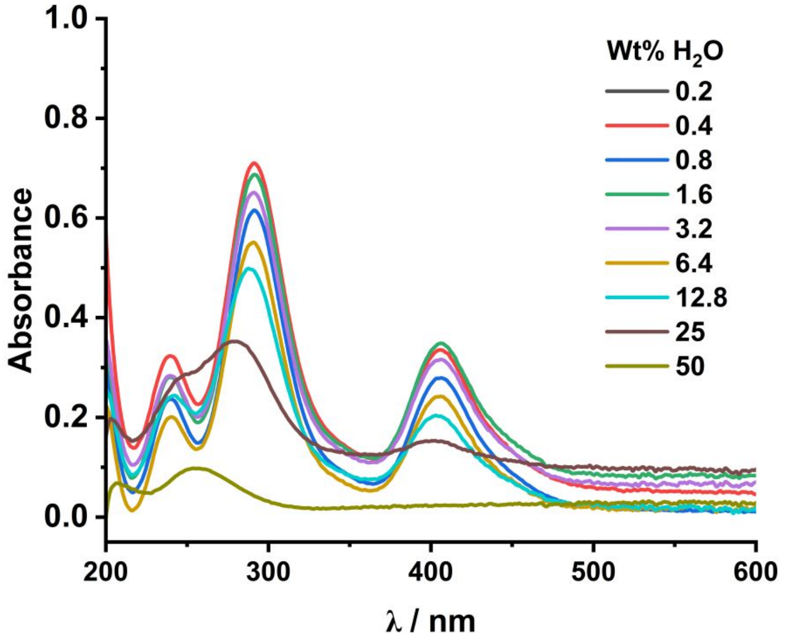

Figure S2: UV-Vis spectra of copper(II) chloride dissolved in ChCl:2EG with varying water content.

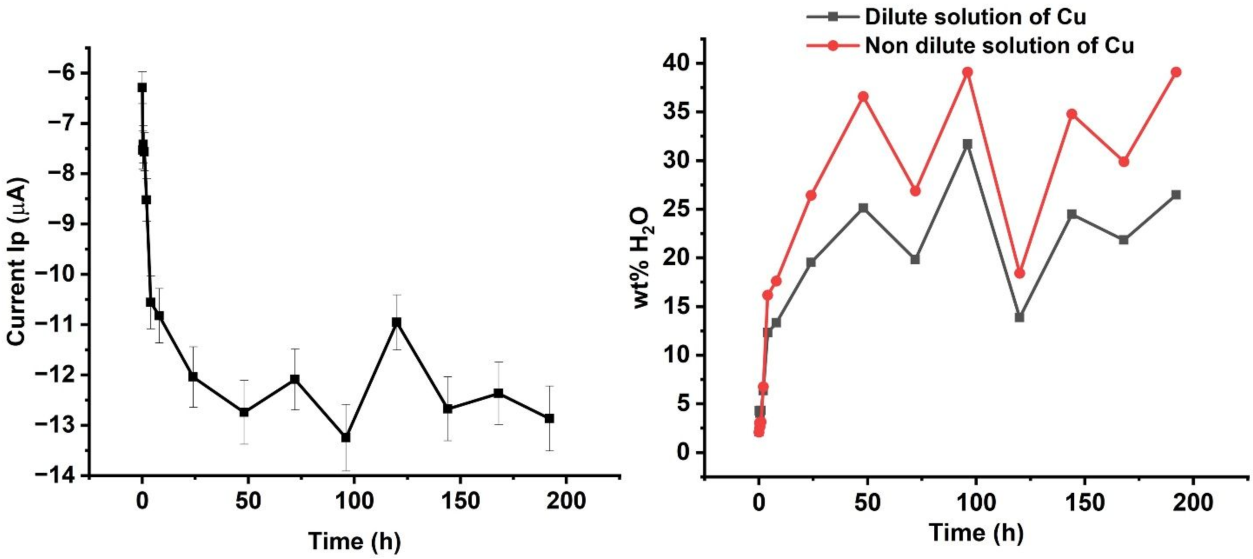

Figure S3: a) Peak current (I_p) of ChCl:2EG measured at different exposure times, corresponding to varying water content. (b) Calculated water weight percentage in ChCl:2EG over time, determined using the I_p calibration curve for diluting $CuCl_2$ and non-diluting $CuCl_2$ with I_p corrected for concentrations (**Figure S1**).

REFERENCES

(1) Bard, A. J.; Faulkner, L. R.; White, H. S. *Electrochemical methods: fundamentals and applications*; John Wiley & Sons, 2022.
